# Chick Chorioallantoic Membrane (CAM) Assay as an *In Vivo* Model to Study the Effect of Newly Identified Molecules on Ovarian Cancer Invasion and Metastasis

**DOI:** 10.3390/ijms13089959

**Published:** 2012-08-10

**Authors:** Noor A. Lokman, Alison S. F. Elder, Carmela Ricciardelli, Martin K. Oehler

**Affiliations:** 1 Research Centre for Reproductive Health, School of Paediatrics and Reproductive Health, Robinson Institute, University of Adelaide, Adelaide, South Australia 5000, Australia; E-Mails: noor.lokman@adelaide.edu.au (N.A.L.); alison.elder@adelaide.edu.au (A.S.F.E.); carmela.ricciardelli@adelaide.edu.au (C.R.); 2 Department of Gynaecological Oncology, Royal Adelaide Hospital, Adelaide, South Australia 5000, Australia

**Keywords:** ovarian cancer, invasion, metastasis, chick chorioallantoic membrane

## Abstract

The majority of ovarian cancer patients present with advanced disease and despite aggressive treatment, prognosis remains poor. Significant improvement in ovarian cancer survival will require the development of more effective molecularly targeted therapeutics. Commonly, mouse models are used for the *in vivo* assessment of potential new therapeutic targets in ovarian cancer. However, animal models are costly and time consuming. Other models, such as the chick embryo chorioallantoic membrane (CAM) assay, are therefore an attractive alternative. CAM assays have been widely used to study angiogenesis and tumor invasion of colorectal, prostate and brain cancers. However, there have been limited studies that have used CAM assays to assess ovarian cancer invasion and metastasis. We have therefore developed a CAM assay protocol to monitor the metastatic properties of ovarian cancer cells (OVCAR-3, SKOV-3 and OV-90) and to study the effect of potential therapeutic molecules *in vivo*. The results from the CAM assay are consistent with cancer cell motility and invasion observed in *in vitro* assays. Our results demonstrate that the CAM assay is a robust and cost effective model to study ovarian cancer cell metastasis. It is therefore a very useful *in vivo* model for screening of potential novel therapeutics.

## 1. Introduction

Ovarian cancer is the most lethal gynecological malignancy. Most patients are diagnosed at an advanced stage when the cancer cells have already metastasized to the abdominal cavity. Ovarian cancer metastasis is characterized by the ability of ovarian cancer cells to detach from the ovary and to adhere and invade the peritoneal cell layer, which lines the organs in the abdominal cavity [1]. The development of new therapies with the aim to disrupt ovarian cancer metastasis requires the *in vivo* study of novel targets and molecules. However, commonly used murine models are costly and require a large number of animals as well as a long experimental time frame. An attractive alternative is the chick chorioallantoic membrane (CAM) assay.

CAM assays have been widely used to study angiogenesis [2], tumor cell invasion and metastasis [3–6]. The CAM model has many advantages, such as (a) the highly vascularized nature of the CAM greatly promotes the efficiency of tumor cell grafting; (b) high reproducibility; (c) simplicity and cost effectiveness, and finally (d) as the CAM assay is a closed system, the half-life of many experimental molecules such as small peptides tends to be much longer in comparison to animal models, allowing experimental study of potential anti-metastatic compounds that are only available in small quantities [4,7]. The CAM is composed of a multilayer epithelium; the ectoderm at the air interface, mesoderm (or stroma) and endoderm at the interface with the allantoic sac [8]. Furthermore, the CAM contains extracellular matrix proteins (ECM) such as fibronectin, laminin, collagen type I and integrin α_ν_β_3_ [9]. The presence of these extracellular matrix proteins mimics the physiological cancer cell environment.

Although the CAM assay is a well established model for studying tumour angiogenesis and invasion in malignancies such as bowel cancer [10,11], glioma [12–14], prostate cancer [15–17], leukemia [18] and osteosarcoma [19], there has only been one study to date that has used a CAM assay to assess ovarian cancer invasion and metastasis [20]. We recently investigated the ovarian cancer-peritoneal cell interaction and identified several novel proteins that may be involved in ovarian cancer metastasis [21,22]. To effectively determine their function, we developed a CAM assay protocol using a range of ovarian cancer cell lines to allow the monitoring of candidate molecules on ovarian cancer cell invasion *in vivo*. The *in vivo* CAM assay data was compared with results from *in vitro* assays.

## 2. Results

### 2.1. Human Ovarian Cancer Cell Motility and Invasion In Vitro

**Figure 1:**
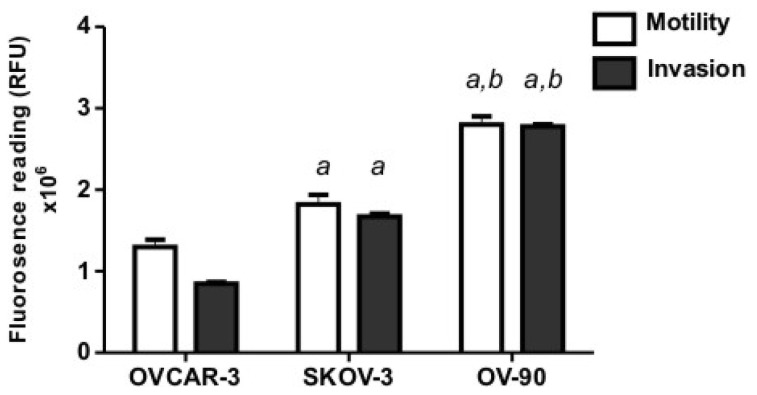
Motility and invasion of human ovarian cancer cell lines (OVCAR-3, SKOV-3 and OV-90) *in vitro*. The fluorescence reading represents the cancer cells that have migrated through the pores or invaded through the extracellular matrix (Geltrex). Data represents the mean ± SEM from two independent experiments performed in quadruplicate. (*a*) Indicates significant difference from OVCAR-3 cells; and (*b*) indicates significant difference from SKOV-3 cells, *p* < 0.05.

We compared the motility and invasion of three ovarian cancer cells (OVCAR-3, SKOV-3 and OV-90) using *in vitro* assays. We found OV-90 cells to be most invasive through an extracellular matrix and migrated faster through 12 μm pores towards a chemo attractant, compared to SKOV-3 and OVCAR-3 cells (Figure 1). OVCAR-3 cells were the least motile and invasive cell line in our study.

### 2.2. Human Ovarian Cancer Cell Invasion into the Chick Chorioallantoic Membrane (CAM)

**Figure 2:**
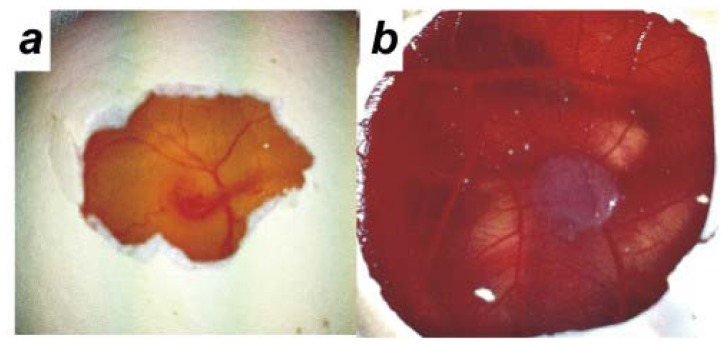
(**a**) Day 3 chick embryo; (**b**) Ovarian cancer cells and matrigel graft on the chick chorioallantoic membrane (CAM) on day 14.

We initially utilized an *ex ovo* method and incubated the chick embryos in plastic weigh boats as described previously [23]. The *ex ovo* method has the advantage of allowing the application of a larger number of matrigel grafts as a wider area of the CAM is accessible. However, the survival rate for the *ex ovo* method was very low and only 10% of embryos survived to day 14. The *in ovo* method had a survival rate of 70% on day 14. In the *in ovo* method, a small window is made in the shell on day 3 of chick embryo development to detach the CAM layer from the egg shell (Figure 2a). Ovarian cancer cells (9 × 10^5^ cells) were mixed with matrigel to form a gel and grafted on top of the CAM of day 11 chick embryos. The chick embryos were incubated with the matrigel grafts until day 14 of development. An example of a matrigel graft on day 14 is shown in Figure 2b.

**Figure 3:**
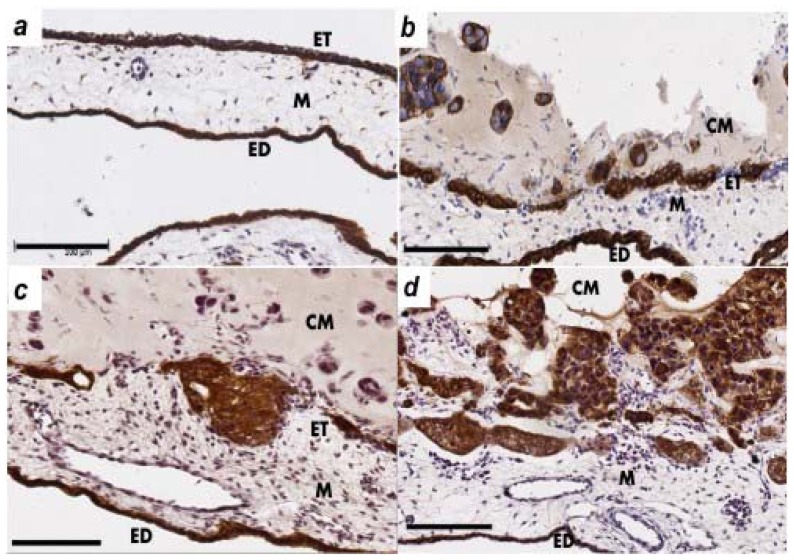
Invasion of ovarian cancer cells in the chick chorioallantoic membrane (CAM). (**a**) Control showing the normal structure of the CAM layers; ectoderm (ET), mesoderm (M) and endoderm (ED); (**b**) OVCAR-3; (**c**) SKOV-3; and (**d**) OV-90 cancer cell matrigel grafts (CM) were placed on top of the ectoderm layer and cancer cell invasion into the CAM mesoderm was assessed in day 14 chick embryos. CAM paraffin sections (6 μm) were immunostained with cytokeratin antibody. Original magnification ×200, scale bar 100 μm.

The CAM layers; ectoderm (ET), mesoderm (M) and endoderm (ED) can be seen in Figure 3a. Cytokeratin immunohistochemistry was used to identify the CAM layer integrity and presence of ovarian cancer cells in the mesodermal layer.

The invasion of the ovarian cancer cells through the ectoderm into the mesoderm was assessed on day 14 of chick embryo development. OVCAR-3 cytokeratin immunohistochemistry showed some damage to the ectoderm layer but minimal invasion into the CAM mesoderm (Figure 3b). The SKOV-3 cells showed invasion into the mesoderm layer and minimal destruction of the ectoderm layer (Figure 3c). OV-90 cells were the most invasive cells in the CAM assay (Figure 3d), which agrees with the results of the *in vitro* assays (Figure 1). Figure 3d shows a large number of OV-90 cells invading into the mesoderm of the CAM, as well as the destruction of the CAM ectoderm layer.

**Figure 4:**
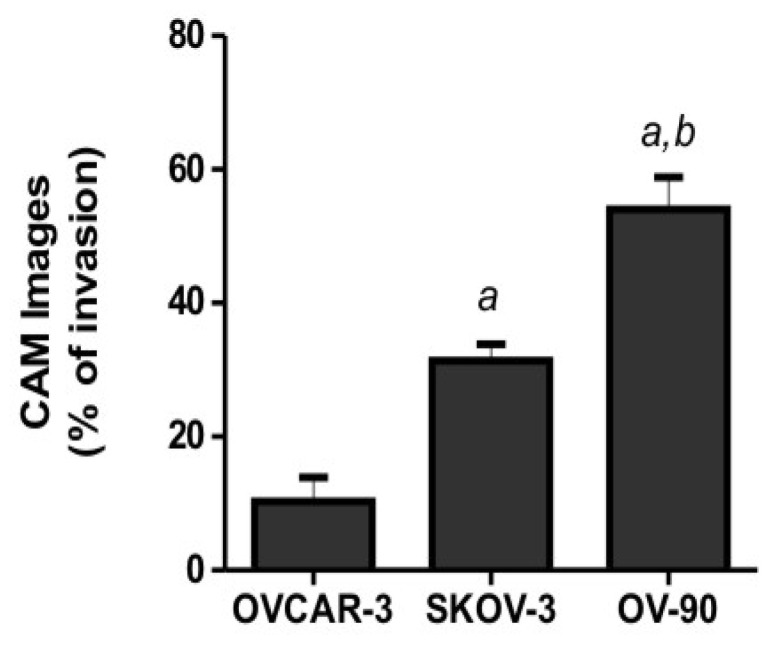
Chick chorioallantoic membrane (CAM) invasion by ovarian cancer cells in day 14 chick embryos. Data generated from 48–60 images from 6 chicken embryos per cell line. Data represents the percentage of images with invasion into the mesoderm, mean ± SEM from two independent experiments. (*a*) Indicates significant difference from OVCAR-3 cells; and (*b*) indicates significant difference from SKOV-3 cells, *p* < 0.05.

Quantitative analysis of ovarian cancer cell invasion into the CAM was performed by determining the number of images (8 to 12 sections per chick embryo) with cancer cell invasion into the CAM mesoderm of day 14 chick embryos. Our results showed a significantly higher invasion of OV-90 and SKOV-3 cells into the CAM mesoderm, when compared with OVCAR-3 cells (Figure 4).

### 2.3. The Effects of Protein a Neutralizing Antibody on OV-90 Cancer Cell Invasion into the CAM

**Figure 5:**
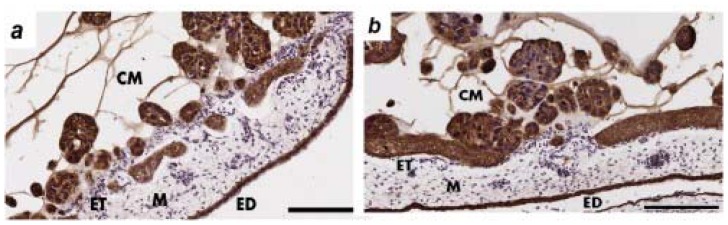
Effects of protein A neutralizing antibody on OV-90 cancer cell invasion into the chick chorioallantoic membrane (CAM). OV-90 cancer cells were mixed with matrigel and (**a**) control anti-mouse IgG (20 μg/mL); or (**b**) neutralizing antibody against protein A (20 μg/mL). CAM paraffin sections (6 μm) were immunostained with a pan-cytokeratin antibody. ET = ectoderm. M = mesoderm. ED = endoderm. CM = cancer cell matrigel grafts. Original magnification ×200, scale bar 100 μm.

We used a neutralizing antibody against one of the novel proteins identified in our previous study investigating ovarian cancer-peritoneal cell interactions [21,22]. OV-90 cancer cells (9 × 10^5^ cells) were mixed with matrigel and the control anti-mouse IgG or the neutralizing antibody against protein A before grafting onto day 11 chick embryos. Neutralizing antibody against protein A effectively inhibited OV-90 cancer cell invasion into the mesoderm of the CAM, compared with the control anti-mouse IgG, where OV-90 cancer cells invaded the mesoderm of the CAM and a destruction of the ectoderm layer was observed (Figure 5).

## 3. Discussion

The CAM assay is a frequently applied model to study ovarian cancer angiogenesis [24–27]. However, there is only one study which has used CAM assays to assess ovarian cancer cell invasion and metastasis [20]. Chang *et al.* described IGROV-1 ovarian cancer cell invasion and metastasis to the posterior CAM and lungs of chick embryos [20]. We have developed a CAM assay protocol to monitor the metastatic properties of ovarian cancer cells (OVCAR-3, SKOV-3 and OV-90) and have successfully used it to study the effect of newly identified molecules *in vivo*. Our results show that the CAM assay is an effective model to study ovarian cancer metastasis. Importantly, our CAM model closely mimics the mode of ovarian cancer metastasis which involves ovarian cancer cell attachment and invasion into the peritoneum. The ectodermal layer of the CAM has many similarities with the peritoneum, which consists of a single layer of mesothelial cells covering the organs in the abdominal cavity.

We observed a higher survival rate with the *in ovo* method in comparison to the *ex ovo* method for monitoring of ovarian cancer cell growth in the chick embryos. Various methods have been used to graft cancer cells in the CAM model; such as collagen onplants [23], plastic rings [19], and matrigel grafts [28]. Furthermore, cancer cells can also be inoculated by dropping the cell suspension on top of the CAM [29], or administered intravenously to study metastasis of cancer cells in the chick embryos [18]. Matrigel is one of the most suitable scaffolds used for implantation and grafting of cancer cells onto the CAM. In our model, ovarian cancer cells and matrigel were mixed with or without neutralizing antibodies before grafting onto the CAM of the chick embryos to assess ovarian cancer cell invasion. The grafting of the matrigel in the CAM model allows continuous visualization of the test site. Moreover, other studies have reported visible and solid tumors on the CAM of chick embryos a few days after cancer cell inoculation [11,19]. We used histological assessment by means of a pan-cytokeratin antibody, to allow the visualization of cancer cells invading into the mesoderm.

The CAM model has been previously employed to assess cancer metastasis [3,29]. In some studies quantitative *alu* PCR was used to assess the presence of metastatic human cancer cells in chick embryo organs [30]. Several studies have compared both CAM assays and mouse models to assess tumor growth and metastasis. Colorectal cancer cells were reported to colonize the CAM similarly to the mouse model [10]. Strojnik *et al.* conducted a study to compare the histological and immunohistochemical characteristics of glioma tumor protein expression in the CAM and an established rat model. They reported a similar profile of proteins expressed in both models [12]. In addition, the CAM model has also been used concurrently with the nude mice model to assess tumor growth of fibrosarcoma (HT1080 cells) and human squamous carcinoma (Hep3 cells) cells [31]. Lyu *et al.* also showed that over expression of urokinase-type plasminogen activator receptor (u-PAR) in Hep3 cells leads to an increase in cancer cell invasion in the CAM model as well as accelerated tumor growth in the SCID mice model [32]. These studies demonstrate the validity of the CAM model for *in vivo* analysis of cancer cell invasion and metastasis.

The CAM model has many advantages. It is cost effective, allows large scale screening and is an easily reproducible *in vivo* model [4,7]. A comparison of the advantages and limitations of the CAM against the mouse model are summarized in Table 1.

The CAM model has also been used in pre-clinical screening to assess the efficacy of drugs and inhibitors on tumor growth. Hagedorn *et al.* reported that treatment of human glioma cells with receptor tyrosine kinase inhibitors inhibited tumor growth in a CAM model [14]. Bekes *et al.*, demonstrated that treatment of prostate cancer (PC3 cells) with u-PA activation blocking antibody mAb-112 significantly inhibited cancer cell invasion in the CAM model [35]. Additionally, the CAM model has been used to test the efficacy of chemotherapy agents (such as doxorubicin) in human leukemia cell lines and has been shown to reduce cancer cell growth in the CAM [18]. An important limitation of the CAM model is the inability to assess cancer–immune cell interactions. Examination of cancer–immune cell interactions requires the use of transgenic ovarian cancer models [36], however, these models are not widely available, are not suitable for high throughput screening, and cannot be used with primary ovarian cancer cells derived from clinical samples. The CAM assay is therefore an attractive model to rapidly assess the effectiveness of novel candidate therapeutic drugs and the *in vivo* inhibition of specific tumor types and subtypes in one consistent model.

We have shown that OV-90 ovarian cancer cells invade into the mesoderm of the CAM within three days of implantation, therefore making the OV-90 CAM model ideal for studying ovarian cancer invasion and metastasis. In contrast, the OVCAR-3 cells showed limited invasion in the CAM over the three days of our assays, and would therefore be suitable for studying the role of molecules that promote ovarian cancer invasion. In conclusion, the CAM model provides a high throughput *in vivo* model for the assessment and evaluation of candidate pro-invasive molecules as well as potential therapeutic targets for ovarian cancer.

## 4. Materials and Methods

### 4.1. Cell Culture

The human ovarian cancer cell lines OVCAR-3, SKOV-3 and OV-90 were obtained from American Type Culture Collection (ATCC, Manassas, VA, USA). All ovarian cancer cell lines were maintained in RPMI 1640 medium supplemented with 4 mM l-glutamine, antibiotics (100 U penicillin G, 100 μg/mL streptomycin sulfate and 0.25 μg/mL amphotericin B), (Sigma-Aldrich, St. Louis, MO, USA). OVCAR-3 and SKOV-3 cells were supplemented with 5% fetal bovine serum (FBS) (Sigma-Aldrich) and OV-90 cells were supplemented with 10% FBS. All cell lines were maintained at 37 °C in an environment of 5% CO_2_.

### 4.2. Cell Motility and Invasion Assays

Cell motility and invasion assays were performed as previously described [37]. Briefly, the ovarian cancer cells (OVCAR-3, SKOV-3 and OV-90) were diluted to a cell concentration of 1 × 10^6^ cells/mL and labeled with calcein-AM (1 μg/mL, Life Technologies, VIC, Australia). Ovarian cancer cells were mixed at room temperature for 1 hour in the dark on an oscillating platform. Ovarian cancer cells (50 μL, 40,000 cells) were loaded on top of uncoated 12 μm filter inserts (Disposable, 96-well plate, ChemoTx, Neuro Probe Inc, Gaitherburg, MD, USA) for motility assays or 12 μm filters coated with Geltrex (0.6 μL/well, 9 mg/mL, Life Technologies) for invasion assays. 10% FBS RPMI media was used as a chemo attractant. The cells were allowed to migrate and invade to the lower chamber for 6 hours. Non-migratory cells on the top of the filter were removed and the fluorescence was measured at 485–520 nm.

### 4.3. Chick Chorioallantoic Membrane Assay (CAM Assay)

Fertilized white leghorn chicken eggs were obtained from Hi-Chick, South Australia, Australia. Eggs were incubated in a MultiQuip Incubator (E2) at 37 °C with 60% humidity. Ethics approval was obtained by the University of Adelaide Animal Ethics Committee. A small window was made in the shell on day 3 of chick embryo development under aseptic conditions. The window was resealed with adhesive tape and eggs were returned to the incubator until day 11 of chick embryo development. On day 11, OVCAR-3, SKOV-3 and OV-90 cell suspensions (9 × 10^5^) were mixed with growth factor reduced matrigel (8.9 mg/mL, BD Biosciences, NSW, Australia) in a total volume of 30 μL. Control anti-mouse IgG (20 μg/mL) (Sigma Aldrich) and neutralizing antibody to protein A (20 μg/mL) (BD Biosciences) were mixed together with the OV-90 cancer cells and matrigel. Matrigel grafts were placed on top of the CAM and eggs were resealed and returned to the incubator for 72 hours until day 14 (n = 6 chicken embryos per cell line). Matrigel grafts with surrounding CAM were harvested from each embryo and fixed with 4% paraformaldehyde for 24 hours and embedded in paraffin. Serial sections (6 μm) were stained with hematoxylin and eosin. Slides were digitally scanned using the NanoZoomer (Hamamatsu Photonics K.K., Japan).

### 4.4. Immunohistochemistry

CAM paraffin sections (6 μm) were incubated on a heat plate at 60 °C for 2 hours. Tissue sections were dewaxed with xylene and ethanol, followed by PBS washes. Antigen retrieval was performed by using 1% protease (Sigma-Aldrich) for 10 minutes on a heat plate at 37 °C. The endogenous peroxidase activity of the sections was quenched with 0.3% H_2_O_2_. Each tissue sample was blocked with 5% goat serum for 30 minutes before incubation with monoclonal mouse anti-human cytokeratin clone AE1/AE3 (1:50, Dako, VIC, Australia) at 4 °C overnight. Subsequently, the tissues sections were incubated sequentially with biotinylated goat anti-mouse (1:400, Dako), followed by streptavidin-HRP conjugated (1:500, Dako) for 1 hour at room temperature. Immunoreactivity was detected using diaminobenzidine/H_2_O_2_ substrate (Sigma-Aldrich). The sections were counterstained with 10% haematoxylin (Sigma-Aldrich), dehydrated and mounted in Pertex (Medite Medizintechnik, Germany). Slides were digitally scanned using the NanoZoomer (Hamamatsu Photonics K.K.). For quantitative analysis of ovarian cancer cell invasion into the mesoderm layer, 8 to 12 CAM images from each embryo were assessed by two independent researchers [16].

### 4.5. Statistical Analysis

All analyses were performed using SPSS 15.0 for Windows software (SPSS, Chicago, IL, USA). The student’s t-test and one-way ANOVA were used to determine statistical significance of ovarian cancer cell motility and invasion *in vitro*, and ovarian cancer cell invasion in the CAM model. Statistical significance was accepted at *p* < 0.05.

## 5. Conclusions

The CAM assay is a robust technique that can be used to monitor invasion of ovarian cancer cell lines and to assess the role of novel molecules and potential therapeutic targets. It is a valuable alternative to murine *in vivo* models for the study of ovarian cancer invasion and metastasis.

## Figures and Tables

**Table 1:** Comparison of the advantages and limitations of the chick chorioallantoic membrane (CAM) and mouse model.

*In vivo* model	Advantages	Reference	Limitations	Reference
**CAM**	Short experimental assay (days)		Short observation period (days)	
	Inexpensive		Cannot examine cancer-immune cell interactions	
	Easily reproducible and high throughput		Rapid morphological changes	[4]
	Closed system—allows assessment of small quantities of therapeutic agents		Limited antibodies to chicken tissues for characterization	[7]
	Naturally immunodeficient	[7]		
	Multiple tests per individual CAM	[4]		
	Allows large scale screening			
	Biology and physiology well known			
	Availability of *in vivo* imaging	[3]		
	Allows direct visualization	[8]		
	Animals do not have to be restrained			
	Can be used with primary human cell lines			
**Mouse**	Longer observation period (weeks to months)		Long experimental length (months to years)	[4]
	Biology and physiology well known, but also complex	[4]	Costly	
	Availability of *in vivo* imaging	[33]	Mature immune system	
	Defined genetic background	[34]	Reproducibility is expensive	
			Large number of animals required	
			Animals have to be restrained	[8]
